# Vision and Hearing Impairments Affecting Activities of Daily Living among Malaysian Older Adults by Gender

**DOI:** 10.3390/ijerph18126271

**Published:** 2021-06-10

**Authors:** Yee Mang Chan, Norhafizah Sahril, Ying Ying Chan, Nor’ Ain Ab Wahab, Norliza Shamsuddin, Muhd Zulfadli Hafiz Ismail

**Affiliations:** 1 Institute for Public Health, National Institutes of Health, Ministry of Health, Shah Alam 40170, Malaysia; norhafizah_s@moh.gov.my (N.S.); chan.yy@moh.gov.my (Y.Y.C.); norain.ab@moh.gov.my (N.A.A.W.); norlizas@moh.gov.my (N.S.); 2 Sector for Biostatistics and Data Repository, National Institutes of Health, Ministry of Health, Shah Alam 40170, Malaysia; m.zulfadli@moh.gov.my

**Keywords:** hearing impairment, vision impairment, ADL disability, older adults

## Abstract

Vision and hearing impairments are common among older adults and can cause undesirable health effects. There are limited studies from low- and middle-income countries exploring gender differences between vision and hearing impairment with Activities of Daily Living (ADL) disability. Therefore, this study aimed to investigate gender differences between vision and hearing impairments with ADL disability among older adults in Malaysia. Cross-sectional data from 3977 respondents aged 60 and above from the Malaysian National Health and Morbidity Survey 2018 were used. We used logistic regression analysis to measure associations between vision and hearing impairments with ADL disability, adjusted for covariates. The prevalence of ADL disability was higher among females than males (*p* < 0.001). The adjusted associations between vision impairment and ADL disability were significant among males (aOR 3.79; 95%CI 2.26, 6.38) and females (aOR 2.66; 95%CI 1.36, 5.21). Similarly, significant adjusted associations were found between hearing impairment and ADL disability among males (aOR 5.76; 95%CI 3.52, 9.40) and females (aOR 3.30; 95%CI 1.17, 9.33). Vision and hearing impairments were significantly associated with ADL disability, with no gender differences identified. Early detection and effective management of vision and hearing impairments are important to prevent ADL disability and improve older adults’ level of independence.

## 1. Introduction

Vision and hearing impairments are common and prevalent among older adults [1,2,3]. The World Health Organization (WHO) estimates that approximately one-third of people above 65 years old are affected by disabling hearing loss in 42 population-based studies [2]. Globally, approximately 185 million people aged 50 years and above are visually impaired [3]. The prevalence of vision impairment among older adults in the Selangor state of Malaysia was 27.3% [4]. More than two-thirds (76.2%) of older adults in the Selangor state of Malaysia had hearing impairment [5]. Both vision and hearing impairments can cause undesirable outcomes on physical and mental health, quality of life and cognitive function among older adults [6,7,8,9,10,11]. A systematic review found that sensory impairment had significant association with quality of life among older adults, with an increase in the severity of hearing and visual impairment resulting in a lower quality of life [11]. Visually impaired older adults in northern Italy had a 2-fold higher probability of having depressive symptoms [6]. In addition, age-related vision and hearing impairments are associated with a higher risk of functional disability [10,12,13]. Reduced ability of older adults to perform activities of daily living can lead to hazardous situations and poor quality of life.

A study conducted among older adults residing in a long-term care facility in four countries (Canada, United States, Finland, Belgium) reported older adults with both vision and hearing impairments were more likely to have highly compromised independence of ADL [13]. Singaporean older adults with either or both vision and hearing impairments were found to be associated with more years of limitation in physical function and in ADL [10]. Older adults with vision impairment also were more likely to report difficulty in ADL—for instance walking, getting outside, getting into and out of a bed or chair, managing medication and preparing meals—than older adults without sensory impairment. Likewise, older adults with hearing impairment were found to be more likely to report similar ADL difficulties than older adults without sensory impairment [12]. A study conducted among older adults from two welfare homes in Malaysia reported older adults with vision impairment experienced more difficulties in ADL [14].

In a study evaluating the association of hearing and vision impairments with negative well-being among community-dwelling older adults, vision impairment was related to reduced functional activity in both genders, but hearing impairment was not [15]. Evidence suggests that although females experience longer life expectancy, they have a higher proportion of disabled years compared to male counterparts [16].

To date, there are limited studies from low- and middle-income countries exploring gender differences in the association between vision and hearing impairments, and ADL disability. Considering the differences between males and females in proportion to disabled years to be lived, this study aimed to investigate gender differences in the association between vision and hearing impairments with ADL disability among older adults in Malaysia.

## 2. Materials and Methods

### 2.1. The National Health and Morbidity Survey 2018 (NHMS 2018)

The NHMS 2018 was a nationally representative health survey in Malaysia for older adults aged 60 years and above. Sample size was calculated using the sample size calculation formula for a prevalence study and was adjusted for the total number of the target population based on the estimated 2017 population, design effect and non-response. Sample size was calculated for each specific objective based on the identified scopes, using a single proportion formula and previous literature estimates. The largest sample size was based on the prevalence of living alone (i.e., 3542). This sample size was chosen to ensure the sample in this study covered all the objectives of NHMS 2018. This ensured the minimum required sample size to meet the desired precision with a 95% confidence interval. Precision was determined based on the 95% confidence interval and the relative standard error. To ensure optimum sample size, the sample was further adjusted for the total number of the target population using 2018 estimates, the design effect of 1.5–4.0 from previous surveys and complex sampling analysis with a 30% non-response rate [17]. A two-stage stratified sampling design was applied. With the assistance from the Department of Statistics Malaysia (DOSM), continuous geographical areas called Enumeration Blocks (EBs) were derived from the entire Malaysia. These EBs formed the sampling frame. Malaysia is a federation that consists of 13 states and three federal territories. The first stage sampling unit was the EB and the second stage sampling unit was Living Quarters (LQs). The selection of EBs was carried out independently within each state (as primary stratum) and within urban or rural areas (as secondary stratum) in accordance with the selection rate determined for each stratum. This was to ensure that the sample size was representative of the national population. A total of 60 EBs in urban areas and 50 EBs in rural areas were selected randomly by the DOSM. All households and persons aged 60 years and above within the selected LQ were invited to join the survey. The target population was older adults aged ≥60 years residing in the community over the past 2 weeks, excluding those in institutions, hostels, hotels or visiting the area. A total of 3977 older adults were interviewed. The detailed methodology of NHMS 2018 can be found elsewhere [17].

### 2.2. Data Collection

A bilingual (Malay and English) structured questionnaire was designed, pre-tested and piloted prior to data collection. Data were collected from July 2018 to September 2018 via a face-to-face data collection method. Data collection was conducted by trained research assistants using mobile devices. For respondents with communication problems, for instance post-stroke, cognitive impairment, speech disabilities or language barrier, proxies assisted in the interview process. Proxies mainly were the family members who knew the respondent best. We obtained written consent form all eligible respondents prior to interviews. For illiterate respondents, verbal consent with thumb prints were obtained in front of a literate witness. Ethical approval for the study was obtained from the Medical Research and Ethics Committee (MREC), Ministry of Health (NMRR-17-2655-39047).

### 2.3. Study Measure

Dependent variable, ADL disability was measured using the Barthel index of activities of daily living [17]. The Barthel index assessed ten items of mobility and self-care: bowel, bladder, grooming, toilet use, feeding, transfer, mobility, dressing, climbing stair and bathing. In this study, the Barthel index was categorized into (i) absence of ADL disability, of which respondents scored a maximum score of 20 and (ii) presence of ADL disability, of which respondents scored less than 20. The Barthel index is one of the commonly used questionnaires to evaluate disability among older adults [18].

The Washington Group Extended Questions Set on Functioning was used to measure the independent variables, hearing and vision impairments [19]. Level of difficulties were grouped into four categories: ‘no difficulty’, ‘some difficulty’, ‘a lot of difficulty’ and ‘cannot do at all’. Vision impairment was defined as a positive response of either ‘a lot of difficulty’ or ‘cannot see at all’. Likewise, hearing impairment was defined as a positive response of either ‘a lot of difficulty’ or ‘cannot hear at all’.

A total of ten covariates were included in this study. Socio-demographic variables included were sex, ethnicity, marital status, education level, individual monthly income and employment status. Ethnicity was classified into ‘Malay’, ‘Chinese’, ‘Indian’, ‘Bumiputra Sabah’, ‘Bumiputra Sarawak’ and ‘Others’. Marital status was grouped into ‘unmarried/separated/divorced/widowed’ or ‘married’. Education level was divided into ‘no formal education’, ‘primary education’, ‘secondary education’ or ‘tertiary education’. Individual monthly income had three categories: ‘<RM1000′, ‘RM1000-RM1999′ or ‘≥RM2000′. Employment status was categorized into ‘yes’ or ‘no’. The presence of chronic diseases was based on self-reported medically diagnosed diabetes mellitus, hypertension and hypercholesterolemia. Self-reported diabetes, hypertension or hypercholesterolemia were defined as being told they have the diseases by a doctor or assistant medical officer. Fall was described based on the question ‘In the last 12 months, have you had a fall?’. The responses were ‘yes’ or ‘no’. Social support was assessed by using the Duke Social Support Index (DSSI) and was categorized into ‘low to fair’, ‘high’ and ‘very high’. Living status was categorized into ‘living alone’ and ‘not living alone’.

### 2.4. Analysis

The prevalence of vision impairment, hearing impairment, ADL disability and covariates was described as frequencies and percentages according to gender for categorical data. In addition, we used logistic regression to investigate differences between male and female groups. Subsequently, logistic regression analysis was used to measure the association between vision and hearing impairments with ADL disability. First, results from an unadjusted model (Model 1) were reported, followed by results from a set of hierarchical models adjusting for the covariates (Model 2 included Model 1 and the socio-demographic covariates, Model 3 included Model 2 and the health-related covariates, and Model 4 included the Model 4 and the social-related covariates). Socio-demographic covariates included in Model 2 were for ethnicity, marital status, education level, employment status and individual monthly income. Health-related covariates included in Model 3 were presence of chronic diseases and falls. Social-related covariates included in Model 4 were living status and social support. Data analyses were conducted using SPSS Statistics 25.0 (IBM Corp., Armonk, NY, U.S.) taking into consideration the complex survey design.

## 3. Results

### 3.1. Sample Characteristics

Table 1 presents the characteristics of study respondents according to gender. A total of 3977 older adults participated in this study, with 1872 male respondents (47.1%) and 2105 female respondents (52.9%). The prevalence of ADL disability was higher among female compared to male respondents (21.2% vs. 12.7%) (*p* < 0.001). Both males and females had similar prevalence of vision and hearing impairments. More than 80% of male respondents were married, and only half of female respondents were married. A majority of the respondents were Malays and received primary education. More than one-third of male respondents were employed, and only 10% of female respondents were employed. Male respondents had a higher individual monthly income level compared to female respondents. Female respondents reported higher prevalence of hypertension and hypercholesterolemia compared to male counterparts. The prevalence of falls was similar between both genders. A majority of the respondents were not living alone.

### 3.2. Association between Vision Impairment and ADL Disability

Table 2 shows the findings of a logistic regression model that describes the associations between vision impairment and ADL disability hierarchically for each gender. In male respondents, the unadjusted OR for vision impairment and ADL disability was 7.35 (95%CI 4.25, 12.72). The inclusion of socio-demographic covariates reduced the magnitude of association between vision impairment and ADL disability (aOR 5.52; 95%CI 3.05, 9.99). The addition of health-related covariates further decreased the magnitude of association to aOR 4.96 (95%CI 2.72, 6.38). In model 4, the addition of social-related covariates further decreased the aOR to 3.79 (95%CI 2.26, 6.38). A similar pattern was seen among female respondents. The unadjusted OR for vision impairment and ADL disability was 4.13 (95%CI 2.22, 7.67). In the fully adjusted model (Model 4), the association had reduced to aOR 2.66 (95%CI 1.36, 5.21).

### 3.3. Association between Hearing Impairment and ADL Disability

Table 3 presents the findings of logistic regression models that describe the associations between hearing impairment and ADL disability hierarchically for each gender. In male respondents the unadjusted OR for hearing impairment and ADL disabililty was 7.97 (95%CI 5.19, 12.23). The inclusion of socio-demographic covariates reduced the magnitude of association between hearing impairment and ADL disability (aOR 6.43; 95%CI 4.18, 9.90). The addition of health-related covariates slightly reduced the magnitude of association to aOR 6.30 (95%CI 4.01-9.90). In model 4, the addition of social-related covariates further decreased the aOR to 5.76 (95%CI 3.52, 9.40). Among female respondents, a comparable trend was found. The unadjusted OR for hearing impairment and ADL disability was 4.57 (95%CI 1.67, 12.50). In the fully adjusted model (Model 4), the association was reduced to aOR 3.30 (95%CI 1.17, 9.33).

### 3.4. Factors Associated with ADL Disability

Respondents with higher educational levels, employment and a higher level of social support were associated with lower odds of having ADL disability, in both genders. Married women were associated with lower odds of having ADL disability. On the other hand, falls were associated with higher odds of ADL disability among males.

## 4. Discussion

The aim of this study was to determine whether the association between vision and hearing impairments with ADL disability differs by gender, considering the differences between males and females in proportion years to be lived, medical conditions and illnesses.

This study found higher prevalence of ADL disability among female respondents than male respondents. This finding is consistent with previous studies [20,21,22,23]. One of the plausible explanations could be the morbidity—mortality paradox. The mortality—morbidity paradox describes that females experience more medical conditions and illnesses than male counterparts over their lifespan, although they have lower mortality rates [24]. Evidence also suggests that although females experience longer life expectancy, they have a higher proportion of disabled years to be lived compared to male counterparts [16].

In this study, vision impairment was significantly associated with ADL disability after adjusting for covariates, in both genders. This is in line with a study conducted among older adults from two welfare homes in Malaysia that reported that older adults with vision impairment experienced more difficulties in ADL [14]. Harada 2008 reported similar findings where vision impairment was found to have a significant association with reduced functional activity in both genders [15]. Similarly, vision impairment was associated with functional decline after adjusting for socio-demographic characteristics and chronic conditions among older females [25]. Older adults with vision impairment also were more likely to report difficulty in walking, getting outside, getting in and out a bed or a chair, managing medication and preparing meals compared to older adults without sensory problems [12]. Therefore, it is important to ensure that vision impairment in older adults is adequately treated or corrected, especially among those with ADL disability, in order to limit the undesirable consequences of vision impairment on older adults’ lives.

Hearing impairment had significant associations with ADL disability in both genders in this study. However, there are contradicting findings in previous studies. A study reported older adults with hearing impairment were more likely to have difficulty in walking, getting outside, getting in and out of a bed or chair, and managing medication compared to older adults without sensory problems [12]. However, another study conducted among rural Japanese older adults found that hearing impairment was not associated with reduced functional activity in both genders [15]. In addition, a study examining the association between hearing impairment and functional decline found no significant association after adjusting for socio-demographic characteristics and medical conditions [25]. The inconsistencies of findings could be due to different methods used in collecting hearing-impairment data. Previous studies assessed hearing impairment using an objective method of either following a standardized protocol with a hand-held audiometer or a pure-tone air-conduction audiometry test in a quiet room. Conversely, this study collected self-reported hearing impairment data.

Respondents with a higher education level were associated with lower odds of having ADL disability, in both genders. This finding is in line with prior studies. A study conducted among older rural Malaysians reported a low educational level as one of the risk factors of ADL disability [26]. A national survey of older adults in Thailand found that respondents with no education experienced a higher risk of disability [27]. In Spain, lower educational status was associated with a higher risk of ADL disability among older populations [28]. Likewise, lack of formal education was associated with a higher risk of ADL disability among Indian older adults [29]. Hence, education levels should be taken into consideration in developing and designing promotional programmes or policies related to vision and hearing impairment or ADL disability among older adults.

This study also found that employed respondents had lower odds of having ADL disability, in both genders. Among Singaporean older adults, a lower likelihood of being employed was associated with higher disability rates [30]. In rural North India, unemployment among older adults was associated with higher disability rates [31]. Perhaps this finding could assist in identifying strategies and interventions to extend the retirement age to ensure that older adults stay active and hence reduce the occurrence of ADL disability.

In both male and female respondents, a higher level of social support was associated with lower odds of ADL disability. In Poland, older adults who did not maintain social contact were twice as likely to have at least one ADL limitation [32]. Extensive social participation was associated with significant reduced risk of functional disability after adjusting for socio-demographic factors, health status and health behavioural factors among older adults in China [33]. Policies and programs providing good social support for older adults are essential to ensure older adults can live healthily and have longer disability-free lives.

This study has several strengths. First, the present study recruited national representative respondents of Malaysian older adults using a proper sampling method whereby a stratified two-stage sampling was carried out to ensure that the sample size was representative of the national population. Secondly, this study included a variety of factors, for instance socio-demographic characteristics, health-related variables and social-related variables in the multivariable analysis, which made the final model robust.

It is important to consider the limitations of this study. First, vision and hearing impairment data were collected using a self-reported method. The data collected using a self-reported method may differ from data collected using objective measurement. Secondly, this study collected cross-sectional data. Hence, we were not able to establish the causal relationship due to the cross-sectional study design.

## 5. Conclusions

In conclusion, both vision and hearing impairments had significant associations with ADL disability among older adults. Both genders appear to show significant associations between vision and hearing impairments with ADL disability. In addition, older adults who were employed and had higher social support and educational levels were shown to have lower odds of having an ADL disability. Early detection and effective management of vision and hearing impairments are important among older adults to prevent ADL disability and improve their level of independence. Hearing and visual screening programs at the primary-care level could help with early detection. Primary healthcare providers should also be empowered with the knowledge and skills to screen, detect and manage vision and hearing impairments. Older adults with hearing and vision impairments could benefit from interventions and rehabilitation programs, such as vision and audiological rehabilitation, which have the potential to reduce the occurrence of ADL disability and lead to a longer disability-free life.

## Figures and Tables

**Table 1:** Characteristics of study respondents according to gender.

Variables	Female, n (%)	Male, n (%)	*p*-Value
^#^ ADL disability			
No Yes	1666 (78.8) 430 (21.2)	1616 (87.3) 253 (12.7)	<0.001 *
Hearing impairment			
No Yes	1986 (93.4) 109 (6.6)	1744 (93.7) 126 (6.3)	0.845
Vision impairment			
No Yes	1981 (95.4) 118 (4.6)	1773 (95.6 96 (4.4)	0.814
Ethnicity			
Malay Chinese Indian Bumiputera Sabah Bumiputera Sarawak Others	1163 (55.2) 341 (24.9) 63 (6.2) 142 (3.8) 78 (3.4) 53 (1.6)	1428 (60.6) 369 (28.2) 63 (6.8) 136 (3.6) 80 (4.1) 61 (2.1)	0.003 * 0.185 0.391 0.010 * 0.091
Marital status			
Unmarried/separated/divorced/widowed Married	1082 (49.8) 1021 (50.2)	269 (13.6) 1602 (86.4)	<0.001 *
Education level			
No formal education Primary Secondary Tertiary	636 (22.8) 968 (44.0) 418 (27.9) 83 (5.3)	170 (5.9) 971 (43.2) 549 (36.7) 182 (14.2)	<0.001 * <0.001 * <0.001 *
Employment status			
No Yes	1865 (89.3) 240 (10.7)	1062 (61.5) 810 (38.5)	<0.001 *
Individual monthly income			
<RM1000 RM1000-1999 ≥RM2000	1605 (72.4) 307 (16.3) 169 (11.3)	914 (43.4) 538 (26.8) 398 (19.7)	<0.001 * <0.001 *
Presence of chronic diseases			
Diabetes Hypertension Hypercholesterolemia	572 (28.8) 1166 (55.1) 906 (45.7)	446 (26.5) 861 (46.9) 670 (37.8)	0.114 <0.001 * <0.001 *
Falls			
No Yes	1792 (85.3) 307 (14.7)	1617 (86.6) 253 (13.4)	0.253
Living status			
Living alone Not living alone	151 (6.5) 1954 (93.5)	144 (7.5) 1728 (92.5)	0.351
Social support			
Low to fair High Very high	714 (43.0) 786 (35.9) 598 (30.1)	547 (27.4) 660 (36.1) 654 (36.5)	0.048 * 0.001 *

Column percentage. * *p* < 0.05. ^#^ “Yes” means score <20 points and “No” means score maximum 20 points using the Barthel Index.

**Table 2:** Unadjusted and adjusted associations between vision impairment and ADL disability.

Variable	Model 1 (OR, 95%CI)		Model 2 (aOR, 95%CI)		Model 3 (aOR, 95%CI)		Model 4 (aOR, 95%CI)	
	Male	Female	Male	Female	Male	Female	Male	Female
Vision impairment								
No Yes	1 7.35 (4.25–12.72)	1 4.13 (2.22–7.67)	1 5.52 (3.05–9.99)	1 3.17 (1.72–5.85)	1 4.96 (2.72–9.03)	1 3.35 (1.85–6.06)	1 3.79 (2.26–6.38)	1 2.66 (1.36–5.21)
Ethnicity								
Malay Chinese Indian Bumiputera Sabah Bumiputera Sarawak Others			1 0.48 (0.32–0.71) 0.27 (0.10–0.74) 0.19 (0.19–0.68) 0.30 (0.30–0.96) 0.45 (0.25–0.81)	1 0.70 (0.42–1.16) 0.73 (0.32-1.67) 0.96 (0.68-1.35) 0.29 (0.12-0.72) 0.51 (0.13-2.02)	1 0.47 (0.31-0.72) 0.21 (0.07-0.64) 0.36 (0.19-0.67) 0.56 (0.30-1.04) 0.41 (0.21-0.77)	1 0.71 (0.43-1.18) 0.76 (0.33-1.77) 0.99 (0.72-1.39) 0.29 (0.12-0.71) 0.54 (0.14-2.06)	1 0.41 (0.27-0.62) 0.15 (0.04-0.51) 0.32 (0.18-0.57) 0.64 (0.35-1.18) 0.44 (0.24-0.81)	1 0.65 (0.40-1.06) 0.74 (0.32-1.70) 0.92 (0.66-1.29) 0.34 (0.14-0.82) 0.57 (0.17-1.92)
Marital status								
Unmarried/separated/divorced/widowed Married			1 0.76 (0.47–1.24)	1 0.56 (0.41–0.77)	1 0.66 (0.40–1.09)	1 0.55 (0.40–0.75)	1 0.74 (0.46–1.20)	1 0.58 (0.42–0.81)
Education level								
No formal education Primary Secondary Tertiary			1 0.37 (0.18–0.76) 0.29 (0.14–0.62) 0.16 (0.05–0.48)	1 0.82 (0.59–1.16) 0.47 (0.28–0.81) 0.12 (0.03–0.54)	1 0.34 (0.17–0.69) 0.27 (0.13–0.57) 0.14 (0.05–0.41)	1 0.84 (0.61–1.15) 0.49 (0.28–0.84) 0.12 (0.03–0.55)	1 0.40 (0.18–0.88) 0.33 (0.15–0.76) 0.18 (0.06–0.56)	1 0.93 (0.68–1.26) 0.51 (0.30–0.87) 0.13 (0.03–0.60)
Employment status								
No Yes			1 0.22 (0.13–0.37)	1 0.34 (0.19–0.60)	1 0.23 (0.14–0.40)	1 0.36 (0.21–0.63)	1 0.23 (0.13–0.39)	1 0.37 (0.21–0.64)
Individual monthly income								
<RM1000 RM1000-1999 ≥RM2000			1 1.04 (0.70–1.56) 0.94 (0.52–1.71)	1 1.0 (0.56–1.81) 1.01 (0.50–2.04)	1 1.01 (0.67–1.51) 0.97 (0.52–1.83)	1 0.96 (0.53–1.74) 1.01 (0.50–2.06)	1 1.13 (0.75–1.70) 1.12 (0.60–2.10)	1 1.08 (0.59–1.95) 1.21 (0.57–2.58)
Presence of chronic diseases								
Diabetes Hypertension Hypercholesterolaemia					1.41 (0.95–2.11) 1.20 (0.77–1.87) 1.53 (1.02–2.30)	1.21 (0.87–1.67) 1.21 (0.80–1.83) 1.16 (0.73–1.87)	1.45 (0.97–2.19) 1.13 (0.69–1.84) 1.50 (1.01–2.23)	1.28 (0.91–1.79) 1.19 (0.76–1.86) 1.21 (0.75–1.96)
Falls								
No Yes					1 2.20 (1.32–3.66)	1 1.61 (1.08–2.38)	1 2.18 (1.31–3.61)	1 1.53 (1.00–2.36)
Living status								
Living alone Not living alone							1 1.43 (0.80–2.57)	1 0.53 (0.31–0.92)
Social support								
Low to fair High Very high							1 0.37 (0.24–0.55) 0.25 (0.17–0.38)	1 0.40 (0.26–0.61) 0.32 (0.22–0.47)

Model 2: Controlled for ethnicity, marital status, education level, employment status, individual monthly income + vision impairment. Model 3: Controlled for ethnicity, marital status, education level, employment status, individual monthly, presence of chronic diseases, falls + vision impairment. Model 4: Controlled for ethnicity, marital status, education level, employment status, individual monthly, presence of chronic diseases, falls, living status, social support + vision impairment.

**Table 3:** Unadjusted and adjusted associations between hearing impairment and ADL disability.

Variable	Model 1 (OR, 95%CI)		Model 2 (aOR, 95%CI)		Model 3 (aOR, 95%CI)		Model 4 (aOR, 95%CI)	
	Male	Female	Male	Female	Male	Female	Male	Female
Hearing impairment								
No Yes	1 7.97 (5.19–12.23)	1 4.57 (1.67–12.50)	1 6.43 (4.18–9.90)	1 3.88 (1.45–10.35)	1 6.30 (4.01–9.90)	1 3.89 (1.48–10.27)	1 5.76 (3.52–9.40)	1 3.30 (1.17–9.33)
Ethnicity								
Malay Chinese Indian Bumiputera Sabah Bumiputera Sarawak Others			1 0.45 (0.30–0.68) 0.22 (0.09–0.56) 0.38 (0.19–0.80) 0.58 (0.33–1.02) 0.16 (0.16–0.90)	1 0.74 (0.47–1.15) 0.65 (0.24–1.72) 0.96 (0.68–1.35) 0.33 (0.13–0.86) 0.53 (0.13–2.16)	1 0.45 (0.30–0.69) 0.17 (0.06–0.50) 0.39 (0.19–0.79) 0.59 (0.32–1.10) 0.39 (0.18–0.83)	1 0.74 (0.47–1.16) 0.69 (0.27–1.77) 1.00 (0.71–1.39) 0.34 (0.13–0.86) 0.15 (0.15–2.18)	1 0.39 (0.25–0.60) 0.11 (0.03–0.38) 0.34 (0.18–0.66) 0.65 (0.33–1.27) 0.45 (0.22–0.89)	1 0.68 (0.44–1.05) 0.65 (0.24–1.79) 0.92 (0.65–1.30) 0.39 (0.16–0.96) 0.59 (0.17–1.03)
Marital status								
Unmarried/separated/divorced/widowed Married			1 0.78 (0.48–1.27)	1 0.56 (0.41–0.76)	1 0.68 (0.42–1.12)	1 0.54 (0.40–0.74)	1 0.78 (0.48–1.27)	1 0.58 (0.42–0.81)
Education level								
No formal education Primary Secondary Tertiary			1 0.39 (0.19–0.80) 0.32 (0.15–0.68) 0.18 (0.06–0.53)	1 0.84 (0.62–1.13) 0.45 (0.27–0.74) 0.12 (0.03–0.56)	1 0.38 (0.18–0.78) 0.32 (0.15–0.68) 0.16 (0.06–0.49)	1 0.85 (0.63–1.14) 0.46 (0.28–0.77) 0.12 (0.03–0.57)	1 0.44 (0.20–1.01) 0.40 (0.17–0.91) 0.22 (0.07–0.67)	1 0.93 (0.70–1.23) 0.48 (0.29–0.80) 0.13 (0.03–0.63)
Employment status								
No Yes			1 0.22 (0.13–0.38)	1 0.35 (0.20–0.62)	1 0.24 (0.14–0.41)	1 0.37 (0.21–0.65)	1 0.23 (0.14–0.40)	1 0.37 (0.21–0.66)
Individual monthly income								
<RM1000 RM1000-1999 ≥RM2000			1 0.94 (0.62–1.43) 0.96 (0.51–1.79)	1 0.89 (0.49–1.64) 0.94 (0.46–1.93)	1 0.92 (0.60–1.39) 0.99 (0.52–1.89)	1 1.08 (0.59–1.97) 1.06 (0.52–2.17)	1 1.01 (0.66–1.53) 1.14 (0.61–2.11)	1 1.20 (0.66–2.18) 1.28 (0.60–2.70)
Presence of chronic diseases								
Diabetes Hypertension Hypercholesterolaemia					1.44 (0.90–2.30) 1.34 (0.85–2.12) 1.43 (0.86–2.37)	1.22 (0.88–1.69) 1.23 (0.85–1.78) 1.10 (0.73–1.66)	1.50 (0.95–2.37) 1.25 (0.77–2.01) 1.43 (0.90–2.26)	1.30 (0.93–1.82) 1.18 (0.79–1.77) 1.16 (0.76–1.78)
Falls								
No Yes					1 2.16 (1.30–3.58)	1 1.57 (1.07–2.31)	1 2.11 (1.26–3.52)	1 1.51 (0.99–2.30)
Living status								
Living along Not living alone							1 1.35 (0.69–2.65)	1 0.60 (0.35–1.04)
Social support								
Low to fair High Very high							1 0.35 (0.22–0.55) 0.23 (0.15–0.37)	1 0.42 (0.28–0.62) 0.31 (0.22–0.45)

Model 2: Controlled for ethnicity, marital status, education level, employment status, individual monthly income + hearing impairment. Model 3: Controlled for ethnicity, marital status, education level, employment status, individual monthly, presence of chronic diseases, falls + hearing impairment. Model 4: Controlled for ethnicity, marital status, education level, employment status, individual monthly, presence of chronic diseases, falls, living status, social support + hearing impairment.

## Data Availability

For data protection purposes, the data used for this study are not publicly available but are available from the Institute for Public Health, Ministry of Health Malaysia upon reasonable request and with permission from the Director General of Health Malaysia.
